# Step counter use in type 2 diabetes: a meta-analysis of randomized controlled trials

**DOI:** 10.1186/1741-7015-12-36

**Published:** 2014-02-27

**Authors:** Shanhu Qiu, Xue Cai, Xiang Chen, Bingquan Yang, Zilin Sun

**Affiliations:** 1 Department of Endocrinology, Zhongda Hospital, Institute of Diabetes, Medical School, Southeast University, Nanjing, P.R. China; 2 Department of Medicine II, Section of Sports Medicine and Rehabilitation, Ulm University, D-89075 Ulm, Germany

**Keywords:** Step counter, Type 2 diabetes, Physical activity, Glycemic control, Meta-analysis, Randomized controlled trial

## Abstract

**Background:**

While step counter use has become popular among type 2 diabetes (T2D) patients, its effectiveness in increasing physical activity (PA) and improving glycemic control has been poorly defined. The aim of this meta-analysis of randomized controlled trials (RCTs) was to evaluate the association of step counter use with PA and glycemic control in T2D patients.

**Methods:**

Articles were identified by searches of PubMed, Web of Science and Cochrane Library from January 1994 to June 2013. RCTs in the English language were included, if they had assessed the effectiveness of step counters as motivating and monitoring tools in T2D patients, with reported changes in steps per day (steps/d) or glycosylated hemoglobin A1c (HbA1c), or both. Data were independently collected by 2 authors and overall estimates were made by a random-effects model.

**Results:**

Of the 551 articles retrieved, 11 RCTs were included. Step counter use significantly increased PA by 1,822 steps/d (7 studies, 861 participants; 95% confidence interval (CI): 751 to 2,894 steps/d) in patients with T2D. Step counter use with a PA goal showed a bigger increase in PA (weighted mean difference (WMD) 3,200 steps/d, 95% CI: 2,053 to 4,347 steps/d) than without (WMD 598 steps/d, 95% CI: −65 to 1,260 steps/d). Further subgroup analysis suggested step counter use with a self-set PA goal (WMD 2,816 steps/d, 95% CI: 1,288 to 4,344 steps/d) made no difference in increasing PA from a 10,000 steps/d goal (WMD 3,820 steps/d, 95% CI: 2,702 to 4,938 steps/d). However, no significant HbA1c change was observed by step counter use (10 studies, 1,423 participants; WMD 0.02%, 95% CI: −0.08% to 0.13%), either with (WMD 0.04%, 95% CI: −0.21% to 0.30%) or without a PA goal (WMD 0.01%, 95% CI: −0.10% to 0.13%).

**Conclusions:**

Step counter use is associated with a significant increase in PA in patients with T2D. However, evidence regarding its effect in improving glycemic control remains insufficient.

**Trial registration:**

PROSPERO CRD42013005236

## Background

Physical activity (PA) is a cornerstone of type 2 diabetes (T2D) management [1]. Increased PA is strongly associated with improvement in insulin sensitivity, glycemic control, weight reduction, and related microvascular and macrovascular complications among T2D [2-6]. However, most patients with T2D do not become regularly active or get adequate PA [7,8], with poor self-efficacy, lack of motivation and surveillance as the main contributing factors [9,10].

Lifestyle interventions to change behavior and promote self-efficacy have been very successful in increasing PA [11-13] and improving health outcomes [11,14,15]. As one of the intervention strategies, the step counter (for example, pedometer or accelerometer) has become popular [15]; it is smart, inexpensive and mainly designed to count the number of steps walked daily. The systematic review by Bravata *et al*. [16] pointed out the effectiveness of step counter use in increasing PA and the importance of a PA goal; however, analysis was carried out not only on the general population, but also on those with arthritis, obesity or diabetes. Their conclusion on the benefit of step counter use in increasing PA in patients with T2D is less robust. Moreover, the contradictory findings in other studies [17,18] raise concerns about its effectiveness as a motivating and monitoring tool in promoting PA in T2D patients. The joint position statement from the American College of Sports Medicine and the American Diabetes Association (2010) recommends patients with T2D to walk more often with a goal in mind (for example, 10,000 steps per day (steps/d)) [1]. The evidence for this recommendation is drawn largely from Bravata *et al*. [16]. It remains questionable whether this encouragement correlates with a significant improvement in PA in patients with T2D.

Well-documented evidence suggests that step counter use decreases blood pressure, lipid profiles and improves the quality of life in patients with T2D [19]; while studies on step counter use for improving glycemic control in patients with T2D give conflicting results [15,19]. Although Bravata *et al*. [16] argued that step counter use was not associated with a decreased fasting serum glucose concentration, its association with chronic glycemic control, as assessed by glycosylated hemoglobin A1c (HbA1c), remains unknown, which is considered to be the mainstay of T2D management.

Thus, it is of great interest to conduct a meta-analysis of RCTs to evaluate the association of step counter use with PA as measured by steps/d, and glycemic control as represented by HbA1c; and to determine the association between PA goal-setting and improvement in PA and glycemic control in patients with T2D.

## Methods

### Data sources and search strategies

The following electronic databases were searched from January 1994 to June 2013: PubMed, Web of Science and Cochrane Library. In consultation with a medical research librarian, the MeSH term “diabetes mellitus” and text words “pedomet*”, “acceleromet*” or “step counter” were combined for search in PubMed, a search strategy that was adapted for other databases (see Additional file 1). The related references of all included articles were collected and hand-searched to make sure no suitable and relevant studies were missed. This meta-analysis is reported with reference to the Preferred Reporting Items for Systematic Reviews and Meta-Analyses (PRISMA) statement [20], and adhered to a registered protocol (PROSPERO CRD42013005236; see Additional file 2).

### Study selection

Inclusion criteria were defined according to the “PICOS” Principle: participants, interventions, comparisons, outcomes and study design. Participants were outpatients who had T2D. Inpatient diabetes, type 1 diabetes, gestational diabetes and pre-diabetes, such as impaired glucose tolerance and fasting glucose, were excluded. Interventions that used step counters as motivating and monitoring tools for increasing PA were included, while those used for monitoring walking speed (for example, steps per minute) or solely for assessing the effects of a lifestyle program on PA were excluded. Interventions were compared to a control arm given the usual care intervention or with step counters used only for counting steps.

RCTs in the English language were eligible for inclusion, if they had included more than 5 participants, and reported changes in steps/d or HbA1c, or both (the primary outcome). Studies were excluded if the data of interest were insufficient or could not be obtained from the authors. Since HbA1c reflects the average blood glucose concentration during the previous 8 to 12 weeks, analyses were limited to step counter use lasting for at least 8 weeks [21].

### Data extraction and quality assessment

Preliminary selection was based on titles and abstracts of retrieved articles. Abstracts without adequate information for inclusion or exclusion criteria were retrieved for full-text evaluation. Two authors (SHQ and XC) selected and independently assessed the studies. Discrepancies were resolved by discussion or consensus.

For each of the relevant articles, extracted data included details of the study population (age and sample size), intervention characteristics (intervention duration, whether a diary was used and a PA goal was set), outcome variables (steps/d or HbA1c, or both), adherence to step counter use and dropout rates. Data extraction was conducted by XC and checked by XC for accuracy or missing information. Quality was assessed independently by 2 authors (SHQ and XC) using the Cochrane Collaboration’s ‘Risk of Bias’ Tool [22], which includes random sequence generation, allocation concealment, blinding of participants and personnel, blinding of outcome assessment, incomplete outcome data and selective reporting. Each item was judged as low, unclear or high risk of bias, according to criteria in the Cochrane Handbook (see Additional file 3) [23].

### Data synthesis and analysis

For trials that reported the standard error (SE) of a mean, the standard deviation (SD) was obtained by multiplying by the square root of the sample size from the appropriate arm. If the 95% confidence interval (CI) was shown instead of an SD, the SD was calculated by dividing the length of the CI by 3.92, and multiplying by the square-root of the sample size (n), provided that n was more than 60. For some trials that compared multiple step counter interventions with a single control group, an approach was applied that combined the multiple intervention arms into a single one to overcome the unit-of-analysis error. If trials had an outcome at 2 time-points, the shorter-term follow-up data were used in the primary analyses. Both final values and change scores from baseline of steps/d and HbA1c were entered in the same meta-analysis, as suggested in the Cochrane Handbook for Systematic Reviews [23]. Data from intention-to-treat (ITT) or per-protocol analyses were entered when available in included studies.

The analyses used Stata Software (Version 11.0, College Station, TX, USA). Summary estimates were analyzed with a random-effects model, which coincides with a fixed-effects model when no heterogeneity is presented [23]. The Cochran Q test was used to assess heterogeneity among the studies, with a threshold *P-*value of 0.1 being considered statistically significant. The degree of inconsistency among trials was estimated by the *I*^2^ statistic, where an *I*^2^ value greater than 50% was considered substantially heterogenic. Heterogeneity was explored using 3 strategies: first, sensitivity analyses were conducted by removing each study individually to check whether it could explain heterogeneity; second, univariate meta-regression analyses helped to assess whether the clinical or methodological variables influenced the outcome estimates; and third, subgroup analyses were performed based on meta-regression analyses and pre-specified relevant study characteristics. Publication bias was detected and assessed by Begg's test and Egger's test.

## Results

### Study characteristics

**Figure 1:**
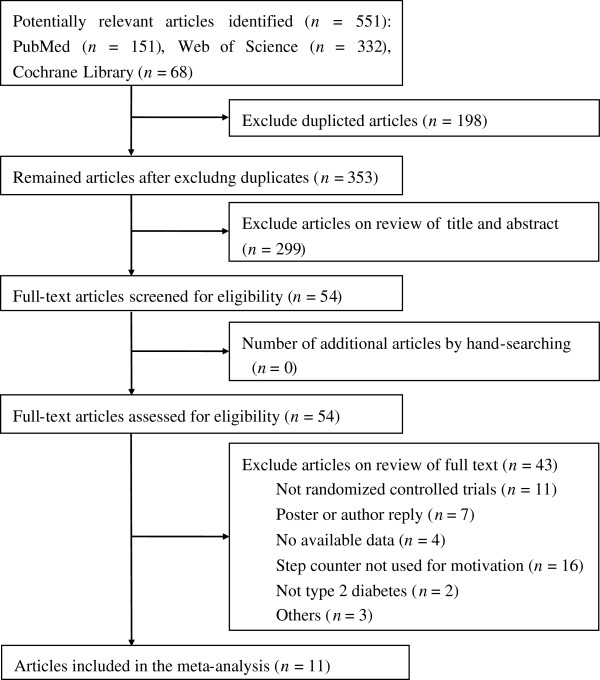
Flow diagram of articles identified.

The databases yielded 551 potentially relevant articles. After careful screening for inclusion and exclusion, 11 RCTs met all the criteria for inclusion in the meta-analysis (Figure 1). Of these, 7 trials reported data of steps/d and 10 trials gave results for HbA1c. Of the trials measuring PA, 3 used the Yamax DigiWalker SW200 pedometer (Yamax Corpo, Tokyo, Japan) [24-26], 1 used the Omron HJ-720ITC pedometer (Omron Healthcare, Inc.; Bannockburn, Illinois, America) [27], with the remaining 3 not giving details [28-30]. Of the trials measuring HbA1c, 1 used the Adams procedure [24], 2 used the DCA 2000 (details not provided) [27,31], 1 used the Tosoh A1c 2.2 Plus Glycohemoglobin Analyzer (Tosoh Medics, Inc.; Foster City, California, America) [26], and the others were unknown [18,25,29,30,32,33]. All 11 trials had been carried out in developed countries: 3 in Belgium, 2 in Britain, 1 in Norway, 2 in America, 1 in Canada and 2 in Australia. The characteristics of these citations are summarized in Table 1.

**Table 1:** Characteristics of the studies included in the meta-analyses

**Source**	**Age mean (SD), y**^**a**^	**Intervention and control description**	**Co-intervention**	**Duration, mo**	**Adherence,%**	**Dropouts,%**
De Greef *et al*. 2011-1^b^[24]	68.3 (8.2)	**Intervention**: a pedometer-based PA program: received a pedometer, set personal goal, instructed to increase self-efficacy and received physical advice.	Diary use; Self-set goal	3	Not stated	2.3
	66.0 (11.1)	**Control**: received usual care				8.3
De Greef *et al*. 2010 [25]	61.3 (6.3)	**Intervention**: a cognitive-behavioral program: received a pedometer; instructed to increase self-efficacy and set new goals; motivated to achieve.	Diary use; Self-set goal	3	75	10
	61.3 (6.9)	**Control**: received usual care				9.5
Tudor-Locke *et al*. 2004 [26]	52.8 (5.7)	**Intervention**: a First Step program: received a pedometer, instructed for self-monitoring and goal-setting, and received postcards for thanks.	Diary use; Self-set goal	4	75	20
	52.5 (4.8)	**Control**: only received postcards for thanks				23.3
Piette *et al*. 2011^e^[27]	55.1 ( 9.4)	**Intervention**: received a telephone-delivered cognitive behavioral therapy, including a pedometer-based PA program, and instructed to progress toward cognitive behavioral therapy goals.	Diary use	9	Not stated	15.7
	56.0 (10.9)	**Control**: received an enhanced usual care				12.6
De Greef *et al*. 2011-2 [28]	62 (9) (total)	**Intervention**: a pedometer-based behavioral modification program with telephone support: received a pedometer, and seven calls for goal-setting, self-monitoring and instructed to increase self-efficacy.	Diary use; 10,000 steps/d	3	Not stated	3.3
		**Control**: received usual care				6.3
Kirk *et al*. 2009^c, e^[29]	62.1 (10.2)	**Intervention**: received a pedometer, a 12-week walking plan and strategies to increase self-efficacy, given physical consultation and follow-up phone calls.	No diary or goal use	6	Not stated	9.1
	59.2 (10.4)	**Control**: received standard care and follow-up phone calls				8.6
Plotnikoff *et al*. 2013^d, e^[30]	61.8 (11.8)	**Intervention**: received a pedometer, PA guidelines and stage-based, print materials for behavior change.	Diary use	12	80	24.9
	61.0 (11.7)	**Control**: received standard PA education materials				10.6
Engel *et al*. 2006 [31]	60.5 (7.34)	**Intervention**: received a pedometer and coaching (which included education, behavior-change strategies and support), instructed to increase self-efficacy, and set steps/d goals.	Diary use; 3,500 to 5,500 steps/d	3	Not stated	12 (total)
	64 (6.76)	**Control**: received coaching only, instructed to increase self-efficacy, and set goals on time spent walking per day				
Bjørgaas *et al*. 2008 [18]	56.4 (11.0)	**Intervention**: received a pedometer, encouraged to increase steps/d and set goals for increasing PA.	Diary use; Increase steps/d	6	Not stated	28
	61.2 (9.7)	**Control**: encouraged to increase the average daily time on walking and set goals				32.4
Andrews *et al*. 2011^e^[32]	60.0 (9.7)	**Intervention**: received intensive diet intervention, a pedometer, and motivating literature; instructed to walk more for five weeks, and then maintain.	Diary use	6	90	1.2
	60.1 (10.2)	**Control**: received intensive diet intervention				0.4
Diedrich *et al*. 2010^e^[33]	56.7 (13.6)	**Intervention**: attended DSMEP, and received a pedometer, a book of Manpo-kei (mainly for motivation).	No diary or goal use	3	Not stated	38 (total)
	54.9 (9.8)	**Control**: attended DSMEP				

DSMEP, Diabetes Self-Management Education Program; PA, physical activity; steps/d, steps per day; SD, standard deviation; mo, month.

^a^In studies with 2 interventions, age data represent combined mean (SD) of each intervention group.

^b^Two intervention group differed in delivery strategy (one was by individual consultation, the other one was by group counseling).

^c^Two intervention group differed in delivery strategy (one was by person, the other one was in written form).

^d^Two intervention group differed in telephone counselling (one was with, the other one was not).

^e^Goals were not specified in those studies.

Four RCTs gave data on adherence to the step counter intervention, with all adherence rates more than 75%. Dropout rates were less than 16% in all but 4 of the 11 studies (Table 1). No major adverse effects related to step counter use, such as musculoskeletal injury, shin soreness or hypoglycemia, were reported. A minor adverse condition was poor health that was not associated with the intervention [30].

Among the 11 included studies, 54.5% (6/11) provided adequate random sequence generation, with 2 trials using a computer generator [24,32], 2 using blocked randomization [27,30], 1 using stratified (gender and age) randomization [25], and 1 using numbered sealed envelopes [29]; 54.5% (6/11) reported proper allocation concealment, with 4 trials using sealed envelopes [24,25,27,29] and 2 using central allocation [30,32]. All studies had blinded assessment of outcomes, and described losses to follow-up and exclusions; 45.5% (5/11) carried out ITT analyses [24,25,28,29,32], whereas 54.5% (6/11) used per-protocol analyses [18,26,27,30,31,33]. The risk of bias assessment for each study is listed in Table 2.

**Table 2:** Bias assessment of each study

**Author, year**	**Random sequence generation**	**Allocation concealment**	**Blinding of participants and personnel**	**Blinding of outcome assessment**	**Incomplete outcome data addressed**	**Selective reporting**
De Greef *et al*. 2011-1 [24]	Low	Low	Low	Low	Low	Low
De Greef *et al*. 2010 [25]	Low	Low	Low	Low	Low	Low
Tudor-Locke *et al*. 2004 [26]	Unclear	Unclear	Low	Low	High	Low
Piette *et al*. 2011 [27]	Low	Low	Low	Low	High	Low
De Greef *et al*. 2011-2 [28]	Unclear	Unclear	Low	Low	Low	Low
Kirk *et al*. 2009 [29]	Low	Low	Low	Low	Low	Low
Plotnikoff *et al*. 2013 [30]	Low	Low	Low	Low	High	Low
Engel *et al*. 2006 [31]	Unclear	Unclear	Low	Low	High	Low
Bjørgaas *et al*. 2008 [18]	Unclear	Unclear	Low	Low	High	Low
Andrews *et al*. 2011 [32]	Low	Low	Low	Low	Low	Low
Diedrich *et al*. 2010 [33]	Unclear	Unclear	Low	Low	High	Low

Summary assessments of the risk of bias for each RCT within studies:

Low risk of bias, low risk of bias for all key domains; unclear risk of bias, unclear risk of bias for one or more key domains; high risk of bias, high risk of bias for one or more key domains.

### Effect on PA

**Figure 2:**
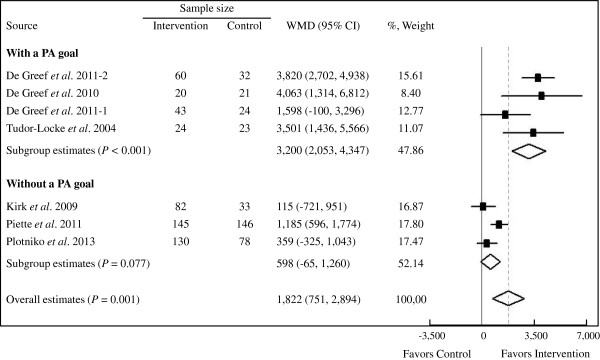
**Forest plot of RCTs investigating step counter use in PA (steps/d) in T2D patients.** The sample size represents the number of participants completing the trials. Summary estimates were analyzed with a random-effects model. CI, confidence interval; PA, physical activity; RCTs, randomized controlled trials; steps/d, steps per day; T2D, type 2 diabetes; WMD, weighted mean difference.

Seven studies (861 participants) comparing step counter use (504 participants) versus control (357 participants) showed that step counter use was associated with a significant increase in PA by 1,822 steps/d (95% CI: 751 to 2,894 steps/d; Figure 2). However, the result was statistically heterogeneous (*P* <0.001, *I*^2^ = 85.9%).

In meta-regression analyses, PA-goal setting partially explained the heterogeneity between these studies, whereas sample size, intervention duration, diary use and study quality could not (see Additional file 4). Subgroup analyses suggested step counter use along with a PA goal (4 studies, 147 participants) significantly increased PA by 3,200 steps/d (95% CI: 2,053 to 4,347 steps/d; *P* for heterogeneity = 0.170, *I*^2^ = 40.3%) compared with the control. Step counter use without a PA goal (3 studies, 357 participants) did not significantly increase the PA (weighted mean difference (WMD) 598 steps/d, 95% CI: −65 to 1,260 steps/d; *P* for heterogeneity = 0.067, *I*^2^ = 63.1%) compared with the control (Figure 2). Further subgroup analysis found no significant difference (*P* = 0.300) between step counter use with a 10,000 steps/d goal (WMD 3,820 steps/d, 95% CI: 2,702 to 4,938 steps/d) or a self-set PA goal (WMD 2,816 steps/d, 95% CI: 1,288 to 4,344 steps/d). Step diary use was also associated with a significant increase in PA (WMD 2,186 steps/d, 95% CI: 962 to 3,411 steps/d); whereas without diary, there was no significant increase (WMD 115 steps/d, 95% CI: −721 to 951 steps/d). When studies were individually removed from this meta-analysis, heterogeneity and WMDs remained unchanged.

No evidence of significant publication bias in the analysis of step counter use was detected by Begg's test (*P* = 0.368) or Egger's test (*P* = 0.147).

### Effect on glycemic control

**Figure 3:**
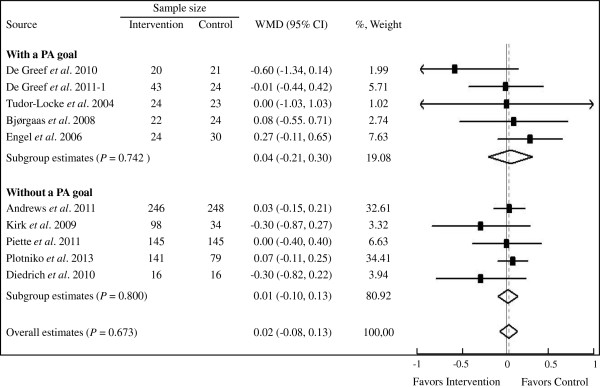
**Forest plot of RCTs investigating step counter use in HbA1c (%) in T2D patients.** The sample size represents the number of participants completing the trials. Summary estimates were analyzed with a random-effects model. CI, confidence interval; HbA1c, glycosylated hemoglobin A1c; PA, physical activity; RCTs, randomized controlled trials; T2D, type 2 diabetes; WMD, weighted mean difference.

Ten studies (1,423 participants) were included in the meta-analysis. The overall, pooled data suggested a non-significant association between step counter use and HbA1c change (WMD 0.02%, 95% CI: −0.08% to 0.13%) compared with the control (Figure 3). No statistical significant heterogeneity was found among the studies (*P* = 0.589, *I*^2^ <1%). Neither step counter use with a PA goal (5 studies, 133 participants) nor without (5 studies, 646 participants) was associated with any significant improvement in HbA1c (WMD 0.04%, 95% CI: −0.21% to 0.30% and WMD 0.01%, 95% CI: −0.10% to 0.13%, respectively) compared with the control (Figure 3). When each study was removed individually from the meta-analysis to evaluate possible individual effects on the summary estimates, heterogeneity and WMDs remained unchanged.

Minor publication bias with under-representation of articles reporting negative effect in HbA1c was noted, as indicated by Begg's test (*P* = 0.107) and Egger's test (*P* = 0.144).

## Discussion

### Summary of the main findings

The results of the meta-analyses show that in patients with T2D, step counter use is associated with a significant increase in PA - a magnitude of 1,822 steps/d. The meta-analyses also show that with a PA goal, step counter use is associated with greater benefit in increasing PA (WMD 3,200 steps/d, 95% CI: 2,053 to 4,347 steps/d) than without it (WMD 598 steps/d, 95% CI: −65 to 1,260 steps/d), indicating that the use of a PA goal is an important predictor of increased PA. Additionally, step counter use with a self-set PA goal makes no difference in increasing PA from a 10,000 step/d goal. However, the analyses do not reveal a conclusive glycemic control benefit of step counter use in T2D patients, regardless of a PA goal or not.

### Interpretation

In accordance with our main results, Bravata *et al*. [16] noted that step counter use was associated with a significant increase of 2,491 steps/d (95% CI: 1,098 to 3,885 steps/d), and setting a steps/d goal (for example, 10,000 steps/d) was an important predictor of increased PA. However, it sounds impractical to recommend patients with T2D to take 10,000 steps/d in the initial period, since the descriptive meta-analysis by Bohannon [34] showed the number of pedometer-assessed steps taken per day by adults aged 65 years or older was much lower than 10,000, and the fact that diabetic patients always show impaired tolerance of PA [35,36]. Considering that 10,000 steps/d goal made no difference from a self-set goal in increasing PA according to our meta-analyses, it is wise and reasonable of patients with T2D to initially set their own steps/d goals, and gradually increase to a recommended higher level (for example, 10,000 steps/d) [1,37]. This study also showed that step diary use was another key motivational factor for increasing PA, which corresponds with the Bravata *et al*. review [16] and an observational study [38].

A cross-sectional study indicated that each SD increment in steps/d (2,609) is associated with a 0.21% lower HbA1c, after adjusting some anthropometric parameters in patients with T2D [39]. Another randomized and stratified study indicated that step counter use increased daily walking, and improved glycemic control by decreasing HbA1c 0.26% in elderly patients with T2D [40]. However, this meta-analysis gave no strong or conclusive evidence to suggest that step counter use could improve glycemic control. There are a number of possible explanations. First, and probably the most important, the baseline HbA1c levels in the included patients with T2D were relatively well controlled (their mean baseline concentrations of HbA1c ranged from 6.64% to 8.0%). To some extent, step counter use can be adopted as an effective strategy for maintaining glycemic control. Second, information on anti-diabetic drug treatment (for example, insulin or sulphonylurea) and dietary intake following step counter intervention was in general poorly recorded, except one study that gave full details on diet [32]. The studies that were included therefore failed to clarify whether the lack of glycemic benefit of step counter use could be attributed to changes in drug dosage or diet. Finally, since the exercise intensity predicted post-intervention HbA1c change to a larger extent than exercise volume in patients with T2D [41], inadequate reporting of walking intensity made it difficult to assess the effectiveness of step counter use in improving glycemic control.

### Strengths and limitations

The strength of this study includes large sample sizes and well-designed RCTs, and is to date the most comprehensive meta-analysis to assess the effectiveness of step counter use in patients with T2D. However, there are several limitations: first, although not indicated by the formal statistical analysis, possibilities remain of publication bias considering that only studies published in the English language were included and 3 electronic databases were searched. Second, high heterogeneity seen in the studies in PA was identified in the meta-analyses and it could not be fully explained by a single related factor. The relatively small number of studies may have contributed to the heterogeneity. Furthermore, high risk of bias due to incomplete outcome data treated with per-protocol analyses could contribute to this heterogeneity. Therefore, better designed RCTs with guidelines for reporting data are urgently needed [42]. Third, the step counters used to measure the steps/d were different or not specified, and the method for determining HbA1c concentration was largely unknown. These could increase the risk of clinical heterogeneity. Fourth, since step counter intervention in all studies was combined with more than one component (for example, step goal, phone call or consultation), it is difficult to clarify the independent contribution of each component. Fifth, this study is limited to the use of HbA1c to analyze glycemic control, as glycemic excursion (variability) is another important marker [43]; however, none of these included trials was examined. Future research should also focus on the glycemic excursion, with regard to glycemic control, when using step counters in patients with T2D. Finally, this study failed to assess the association between step counter use and other cardiometabolic risk factors, such as blood pressure, lipids and lipoproteins.

## Conclusions

In conclusion, step counter use leads to a significant increase in PA in patients with T2D, which is comparable to that given in the previous report [16]. The use of a PA goal is an important predictor of increased PA, and it seems initially better to use a self-set PA goal. However, evidence regarding the effect of step counter use in improving glycemic control remains insufficient in this meta-analysis. More research with better detailed PA goals, and focusing more on medication use, walking intensity and glycemic excursion, is warranted.

## Abbreviations

CI: Confidence interval; DSMEP: Diabetes Self-Management Education Program; HbA1c: Glycosylated hemoglobin A1c; ITT: Intention-to-treat; mo: month; PA: Physical activity; PRISMA: Preferred reporting items for systematic reviews and meta-analyses; RCT: Randomized controlled trial; SD: Standard deviation; SE: Standard error; steps/d: Steps per day; T2D: Type 2 diabetes; WMD: Weighted mean difference.

## Competing interests

The authors declare that they have no competing interests.

## Authors’ contributions

SHQ and XC carried out the study, participated in the data collection and analysis, and drafted the manuscript. XC participated in the data collection and data check. SHQ and ZLS conceived of the study, and ZLS helped to draft the manuscript. BQY helped to improve the English language and gave suggestions to this manuscript. All authors approved the final version of the manuscript.

## Pre-publication history

The pre-publication history for this paper can be accessed here:

http://www.biomedcentral.com/1741-7015/12/36/prepub

## Supplementary Material

Additional file 1 Search strategies. Click here for file

Additional file 2 A registered protocol (PROSPERO CRD42013005236). Click here for file

Additional file 3 Criteria for judging risk of bias of each item using the ‘Risk of bias’ assessment tool. Click here for file

Additional file 4 Univariate meta-regression analyses in PA by different covariates in patients with T2D. Click here for file
