# Supplementary material for: Step counter use in type 2 diabetes: a meta-analysis of randomized controlled trials
Source: BMC Med. 2014 Feb 27;12:36. doi: 10.1186/1741-7015-12-36 (PMC4016223; doi:10.1186/1741-7015-12-36)
Supplement: Additional file 1 — Search strategies. [file 1741-7015-12-36-S1.pdf]

Search strategy in PubMed

# 1	(“Diabetes mellitus“[Mesh]) AND acceleromet*[Text Word]<br>Filter: Publication date from 1994/01/01 to 2013/06/30	84
# 2	("Diabetes Mellitus"[Mesh]) AND pedomet*[Text Word]<br>Filter: Publication date from 1994/01/01 to 2013/06/30	73
# 3	("Diabetes Mellitus"[Mesh]) AND step counter[Text Word]<br>Filter: Publication date from 1994/01/01 to 2013/06/30	3
# 4	#1 OR #2 OR #3	154
# 5	#4<br>Filter: English	151

Search strategy in Cochrane Library

# 1	pedomet*[All Text] AND diabetes[All Text]<br>Limit: Date=1994/01/01 to 2013/06/30	88
# 2	acceleromet*[All Text] AND diabetes[All Text]<br>Limit: Date=1994/01/01 to 2013/06/30	64
# 3	step counter[All Text] AND diabetes[All Text]<br>Limit: Date=1994/01/01 to 2013/06/30	72
# 4	#1 OR #2 OR #3	189
# 5	# 4 in database of “The Cochrane Central Register of Controlled Trials”[†]	68
# 6	# 5<br>Limit: Language=English (by hand)	68

[†]Cochrane Library consists of Cochrane Database of Systematic Reviews (CDSR), Database of Abstracts of Reviews of Effects (DARE), The Cochrane Central Register of Controlled Trials (CENTRAL), The Cochrane Methodology Register (CMR), Health technology assessment database (HTAD), NHS Economic evaluation database (NHSEED) and About the Cochrane Collaboration (ABOUT). Considering that different databases in Cochrane Library represent different types of studies, and clinical trials were of our interest, therefore, we chose the database of CENTRAL at last step for further screening.

Search strategy in Web of Science (SCI-EXPANDED, SSCI, A&HCI, CPCI-S, CPCI-SSH, CCR-EXPANDED, IC)

# 1	TS=diabetes<br>Databases=SCI-EXPANDED, SSCI, A&HCI, CPCI-S, CPCI-SSH, CCR-EXPANDED, IC<br>Timespan=1994-2013	289 643
# 2	TS=acceleromet*<br>Databases=SCI-EXPANDED, SSCI, A&HCI, CPCI-S, CPCI-SSH, CCR-EXPANDED, IC<br>Timespan=1994-2013	17 098
# 3	#2 AND #1	193
# 4	TS=pedomet*<br>Databases=SCI-EXPANDED, SSCI, A&HCI, CPCI-S, CPCI-SSH, CCR-EXPANDED, IC<br>Timespan=1994-2013	1911
# 5	#4 AND #1	153
# 6	TS=step counter<br>Databases=SCI-EXPANDED, SSCI, A&HCI, CPCI-S, CPCI-SSH, CCR-EXPANDED, IC<br>Timespan=1994-2013	2920
# 7	#6 AND #1	31
# 8	#7 OR #5 OR #3	351
# 9	# 8 AND Language=(English)	348
# 10	By hand<br># 9 using Timespan = 1994/01/01 to 2013/06/30	 332
