# Supplementary material for: Step counter use in type 2 diabetes: a meta-analysis of randomized controlled trials
Source: BMC Med. 2014 Feb 27;12:36. doi: 10.1186/1741-7015-12-36 (PMC4016223; doi:10.1186/1741-7015-12-36)
Supplement: Additional file 2 — A registered protocol (PROSPERO CRD42013005236). [file 1741-7015-12-36-S2.pdf]

PROSPERO International prospective register of systematic reviews

Review title and timescale

1 Review title

Give the working title of the review. This must be in English. Ideally it should state succinctly the interventions or exposures being reviewed and the associated health or social problem being addressed in the review.

Step counter use in type 2 diabetes: a meta-analysis of randomized controlled trials

2 Original language title

For reviews in languages other than English, this field should be used to enter the title in the language of the review. This will be displayed together with the English language title.

3 Anticipated or actual start date

Give the date when the systematic review commenced, or is expected to commence.

06/08/2013

4 Anticipated completion date

Give the date by which the review is expected to be completed.

31/10/2013

5 Stage of review at time of this submission

Indicate the stage of progress of the review by ticking the relevant boxes. Reviews that have progressed beyond the point of completing data extraction at the time of initial registration are not eligible for inclusion in PROSPERO. This field should be updated when any amendments are made to a published record.

The review has not yet started ×

Review stage	Started	Completed
Preliminary searches	Yes	Yes
Piloting of the study selection process	Yes	Yes
Formal screening of search results against eligibility criteria	Yes	Yes
Data extraction	Yes	Yes
Risk of bias (quality) assessment	Yes	Yes
Data analysis	Yes	Yes

Provide any other relevant information about the stage of the review here.

Review team details

6 Named contact

The named contact acts as the guarantor for the accuracy of the information presented in the register record.

Shanhu Qiu

7 Named contact email

Enter the electronic mail address of the named contact.

tigershanhu@126.com

8 Named contact address

Enter the full postal address for the named contact.

Xinmofan Road No.3, Nanjing, China

9 Named contact phone number

Enter the telephone number for the named contact, including international dialing code.

0086 025 83285150

10 Organisational affiliation of the review

Full title of the organisational affiliations for this review, and website address if available. This field may be completed as 'None' if the review is not affiliated to any organisation.

Zhongda Hospital, Southeast University, China

Website address:
http://www.njzdyy.com

11 Review team members and their organisational affiliations

Give the title, first name and last name of all members of the team working directly on the review. Give the organisational affiliations of each member of the review team.

Title	First name	Last name	Affiliation
Mr	Shanhu	Qiu	
Ms	Xue	Cai	
Professor	Zilin	Sun	
Mrs	Xiang	Chen	
	Bingquan	Yang	

12 Funding sources/sponsors

Give details of the individuals, organizations, groups or other legal entities who take responsibility for initiating, managing, sponsoring and/or financing the review. Any unique identification numbers assigned to the review by the individuals or bodies listed should be included.

This study was funded by the Key Program of Jiangsu Natural Science Foundation (BK 2010087).

13 Conflicts of interest

List any conditions that could lead to actual or perceived undue influence on judgements concerning the main topic investigated in the review.

Are there any actual or potential conflicts of interest?

None known

14 Collaborators

Give the name, affiliation and role of any individuals or organisations who are working on the review but who are not listed as review team members.

Title	First name	Last name	Organisation details
Mr	Bingquan	Yang	

Review methods

15 Review question(s)

State the question(s) to be addressed / review objectives. Please complete a separate box for each question.

How does the step counter, which was used as a motivational and monitoring tool for increasing physical activity in the lifestyle intervention program, impact on physical activity and glycemic control among type 2 diabetes patients when compared with being used only for counting steps or not being used in the control arm?

16 Searches

Give details of the sources to be searched, and any restrictions (e.g. language or publication period). The full search strategy is not required, but may be supplied as a link or attachment.

Relevant articles were identified by electronic searches of PubMed, Web of Science and Cochrane Library databases from 1994 to June 2013. In consultation with a medical research librarian, MeSH term as “diabetes mellitus” and text words as “pedomet*” or “acceleromet*” or “step counter” were combined for search in Pubmed, and the search strategy was adapted for other databases. English-language randomized controlled trials were eligible for inclusion.

17 URL to search strategy

If you have one, give the link to your search strategy here. Alternatively you can e-mail this to PROSPERO and we will store and link to it.

I give permission for this file to be made publicly available

Yes

18 Condition or domain being studied

Give a short description of the disease, condition or healthcare domain being studied. This could include health and wellbeing outcomes.

19 Participants/population

Give summary criteria for the participants or populations being studied by the review. The preferred format includes details of both inclusion and exclusion criteria.

Inclusion: Outpatients with Type 2 diabetes Exclusion: Type 1 diabetes, Gestational diabetes, Inpatients with Type 1, Type 2 or Gestational diabetes, or outpatients with impaired glucose tolerance.

20 Intervention(s), exposure(s)

Give full and clear descriptions of the nature of the interventions or the exposures to be reviewed

Inclusion: Step counters, such as pedometers and accelerometers, were used as motivational and step counting tools for increasing physical activity in a lifestyle intervention program, in which sometimes also contained step goals, diaries, counseling and education. A reported change in number of steps per day (steps/d) or glycosylated hemoglobin A1c (HbA1c), or both, should also be necessary. Exclusion: Step counters were used only for counting steps or monitoring walking speed (such as steps per minute) in the intervention program. Or there was no reported change in steps/d or HbA1c.

21 Comparator(s)/control

Where relevant, give details of the alternatives against which the main subject/topic of the review will be compared (e.g. another intervention or a non-exposed control group).

We will include control groups with usual care (no step counter intervention), or with step counters only for counting steps.

22 Types of study to be included initially

Give details of the study designs to be included in the review. If there are no restrictions on the types of study design eligible for inclusion, this should be stated.

We will include randomized controlled trials to assess the effectiveness of a step counter use in increasing physical activity and improving glycemic control.

23 Context

Give summary details of the setting and other relevant characteristics which help define the inclusion or exclusion criteria.

24 Primary outcome(s)

Give the most important outcomes.

The most important outcomes are steps/d and HbA1c.

Give information on timing and effect measures, as appropriate.

Steps/d will be calculated mainly by pedometer.

25 Secondary outcomes

List any additional outcomes that will be addressed. If there are no secondary outcomes enter None.

None.

Give information on timing and effect measures, as appropriate.

26 Data extraction, (selection and coding)

Give the procedure for selecting studies for the review and extracting data, including the number of researchers involved and how discrepancies will be resolved. List the data to be extracted.

27 Risk of bias (quality) assessment

State whether and how risk of bias will be assessed, how the quality of individual studies will be assessed, and whether and how this will influence the planned synthesis.

Data from included RCTs will be checked for: missing data and internal data consistency. Quality assessment form will be developed to assess the risk of bias of included studies, which form will be based on the template suggested by Centre for Review and Dissemination (CRD) for systematic reviews in their guidelines. All of these will be independently done by 2 authors. Discrepancies over the risk of bias in particular studies will be resolved by discussion or consensus. Sensitivity analyses will be applied to test the effect of removing each study in the synthesis.

28 Strategy for data synthesis

Give the planned general approach to be used, for example whether the data to be used will be aggregate or at the

level of individual participants, and whether a quantitative or narrative (descriptive) synthesis is planned. Where appropriate a brief outline of analytic approach should be given.
We will provide a descriptive synthesis from the eligible studies including study characteristics (authors and publication year), numbers of participants, and details of the intervention and control groups. We will also provide summaries of intervention effects (Steps/d and HbA1c) for each study by calculating wighted mean differences. All summary estimates were analyzed with a random-effects model. Cochran Q test was used to assess heterogeneity among studies, with a threshold P value of 0.1 being considered statistically significant. The degree of inconsistency among trails was estimated using the I-square test, where an I-square value greater than 50% was considered to be of substantial heterogeneity. Heterogeneity was explored with three strategies. First, sensitivity analyses were conducted by removing each study individually to check whether a particular study could explain heterogeneity. Second, univariate meta-regression analyses were performed to assess whether the clinical or methodological variables could influence the outcome estimates. Third, subgroup analyses were carried out based on meta-regression analyses and prespecified relevant study characteristics. Publication bias was detected and evaluated by Begg's test and Egger's test.

29 Analysis of subgroups or subsets
Give any planned exploration of subgroups or subsets within the review. 'None planned' is a valid response if no subgroup analyses are planned.
We anticipate that subgroup analyses will be done based on meta-regression analyses and prespecified relevant study characteristics, such as sample size, intervention duration, diary use, goal setting and the quality score.

Review general information

30 Type of review
Select the type of review from the drop down list.
Intervention

31 Language
Select the language(s) in which the review is being written and will be made available, from the drop down list. Use the control key to select more than one language.
English

Will a summary/abstract be made available in English?
Yes

32 Country
Select the country in which the review is being carried out from the drop down list. For multi-national collaborations select all the countries involved. Use the control key to select more than one country.
China

33 Other registration details
List places where the systematic review title or protocol is registered (such as with he Campbell Collaboration, or The Joanna Briggs Institute). The name of the organisation and any unique identification number assigned to the review by that organization should be included.

34 Reference and/or URL for published protocol
Give the citation for the published protocol, if there is one.
Give the link to the published protocol, if there is one. This may be to an external site or to a protocol deposited with CRD in pdf format.

I give permission for this file to be made publicly available
Yes

35 Dissemination plans
Give brief details of plans for communicating essential messages from the review to the appropriate audiences.
Do you intend to publish the review on completion?
Yes

36 Keywords

Give words or phrases that best describe the review. (One word per box, create a new box for each term)

step counter

type 2 diabetes

physical activity

glycemic control

meta-analysis

randomized controlled trial

37 Details of any existing review of the same topic by the same authors

Give details of earlier versions of the systematic review if an update of an existing review is being registered, including full bibliographic reference if possible.

38 Current review status

Review status should be updated when the review is completed and when it is published.

Completed but not published

28/03/2014

39 Any additional information

Provide any further information the review team consider relevant to the registration of the review.

40 Details of final report/publication(s)

This field should be left empty until details of the completed review are available.
Give the full citation for the final report or publication of the systematic review.
Give the URL where available.
