# Supplementary material for: Step counter use in type 2 diabetes: a meta-analysis of randomized controlled trials
Source: BMC Med. 2014 Feb 27;12:36. doi: 10.1186/1741-7015-12-36 (PMC4016223; doi:10.1186/1741-7015-12-36)
Supplement: Additional file 3 — Criteria for judging risk of bias of each item using the ‘Risk of bias’ assessment tool. [file 1741-7015-12-36-S3.pdf]

Criteria for judging risk of bias of each item using the 'Risk of bias' assessment tool*

	Low risk	Unclear risk	High risk
**Random sequence generation**	1. Simple randomization;<br>2. Restricted randomization;<br>3. Stratified randomization	Insufficient information to judge 'Low risk' or 'High risk'	1. Sequence generated by odd, date of birth, or by some rule based on date of admission or based on hospital or clinic record number;<br>2. Allocation by judgement of the clinician or by preference of the participant, or by availability of the intervention;<br>3. Allocation based on the results of a laboratory test or a series of tests.
**Allocation concealment**	1. Central allocation (including telephone, web-based and pharmacy-controlled randomization);<br>2. Sequentially numbered drug containers of identical appearance;<br>3. Sequentially numbered, opaque, sealed envelopes.	Insufficient information to judge 'Low risk' or 'High risk'	1. Using an open random allocation schedule;<br>2. Assignment envelopes were used without appropriate safeguards;<br>3. Alternation or rotation<br>4. Date of birth;<br>5. Case record number;<br>6. Any other explicitly unconcealed procedure.
**Blinding of participants and personnel**	1. No blinding or incomplete blinding, but the review authors judge that the outcome is not likely to be influenced by lack of blinding;<br>2. Blinding of participants and key study personnel ensured, and unlikely that the blinding could have been broken.	1. Insufficient information to judge 'Low risk' or 'High risk';<br><br>2. The study did not address this outcome.	1. No blinding or incomplete blinding, and the outcome is likely to be influenced by lack of blinding;<br>2. Blinding of key study participants and personnel attempted, but likely that the blinding could have been broken, and the outcome is likely to be influenced by lack of blinding.
**Blinding of outcome assessment**	1. No blinding of outcome assessment, but the review authors judge that the outcome measurement is not likely to be influenced by lack of blinding;<br>2. Blinding of outcome assessment ensured, and unlikely that the blinding could have been broken.	1. Insufficient information to judge 'Low risk' or 'High risk';<br><br>2. The study did not address this outcome.	1. No blinding of outcome assessment, and the outcome measurement is likely to be influenced by lack of blinding;<br>2. Blinding of outcome assessment, but likely that the blinding could have been broken, and the outcome measurement is likely to be influenced by lack of blinding.
**Incomplete outcome data**	1. No missing outcome data;<br>2. Reasons for missing outcome data unlikely to be related to true outcome;<br>3. Missing outcome data balanced in numbers across intervention groups, with similar reasons for missing data across groups;<br>4. For dichotomous outcome data, the proportion of missing outcomes compared with observed event risk not enough to have a clinically relevant impact on the intervention effect estimate;<br>5. For continuous outcome data, plausible effect size among missing outcomes not enough to have a clinically relevant impact on observed effect size;<br>6. Missing data have been imputed using appropriate methods.	1. Insufficient reporting of attrition/exclusions to judge 'Low risk' or 'High risk';<br><br>2. The study did not address this outcome.	1. Reason for missing outcome data likely to be related to true outcome, with either imbalance in numbers or reasons for missing data across intervention groups;<br>2. For dichotomous outcome data, the proportion of missing outcomes compared with observed event risk enough to induce clinically relevant bias in intervention effect estimate;<br>3. For continuous outcome data, plausible effect size (difference in means or standardized difference in means) among missing outcomes enough to induce clinically relevant bias in observed effect size;<br>4. 'As-treated' analysis done with substantial departure of the intervention received from that assigned at randomization;<br>5. Potentially inappropriate application of simple imputation.

**Selective reporting**	1. The study protocol is available and all of the study's pre-specified outcomes that are of interest in the review have been reported in the pre-specified way;<br>2. The study protocol is not available but it is clear that the published reports include all expected outcomes, including those that were pre-specified.	Insufficient information to judge 'Low risk' or 'High risk'.	1. Not all of the study's pre-specified primary outcomes have been reported;<br>2. One or more primary outcomes is reported using measurements, analysis methods or subsets of the data that were not pre-specified;<br>3. One or more reported primary outcomes were not pre-specified (unless clear justification for their reporting is provided, such as an unexpected adverse effect);<br>4. One or more outcomes of interest in the review are reported incompletely so that they cannot be entered in a meta-analysis;<br>5. The study report fails to include results for a key outcome that would be expected to have been reported for such a study.

*The criteria for judging risk of bias are adapted from: **Cochrane Handbook for Systematic Reviews of Interventions Version 5.1.0 [updated March 2011]** [http://handbook.cochrane.org/]
