# Supplementary material for: Step counter use in type 2 diabetes: a meta-analysis of randomized controlled trials
Source: BMC Med. 2014 Feb 27;12:36. doi: 10.1186/1741-7015-12-36 (PMC4016223; doi:10.1186/1741-7015-12-36)
Supplement: Additional file 4 — Univariate meta-regression analyses in PA by different covariates in patients with T2D. [file 1741-7015-12-36-S4.pdf]

Univariate meta-regression analyses in PA by different covariates in patients with T2D

Covariates	No. of studies	Regression coef [a]	95% CI	*P*	Adjusted R^2 (%) [b]	I^2_{res} (%) [c]
Goal setting	7	2,628.00	985.61 to 4,270.39	0.009	86.91	86.91
Sample size	7	-9.91	-26.92 to 7.10	0.195	15.14	15.14
Intervention duration	7	-282.03	-752.92 to 188.87	0.184	19.42	19.42
Diary use	7	2,090.99	-2,071.58 to 6,253.55	0.253	8.63	8.63
Study quality	7	-76.91	-1,995.48 to 1,841.65	0.922	-24.86	-24.86

Abbreviation: PA, physical activity; T2D, type 2 diabetes; regression coef, regression coefficient; CI, confidence interval.

[a] The regression coefficient obtained from the meta-regression analysis describes how the outcome variable (PA (steps per day)) changes with a unit increase in the explanatory variable.

[b] Proportion of between-study variance explained.

[c] % residual variation due to heterogeneity.
